# Report of 12-months efficacy and safety of intravitreal fluocinolone acetonide implant for the treatment of chronic diabetic macular oedema: a real-world result in the United Kingdom

**DOI:** 10.1038/eye.2016.301

**Published:** 2017-01-20

**Authors:** F Alfaqawi, P L Lip, S Elsherbiny, R Chavan, A Mitra, B Mushtaq

**Affiliations:** 1 Department of Ophthalmology, Birmingham and Midland Eye Centre, City Hospital, Birmingham, West Midlands, UK

## Abstract

***Purpose*:**

To report the 12-months visual and anatomical outcomes of chronic diabetic macular oedema (DMO) treated with ILUVIEN in a real-world clinical practice in a single tertiary referral centre.

***Method*:**

Retrospective data collection and analysis of consecutive 28 eyes of 23 diabetic patients received ILUVIEN implant for refractory DMO. Standard assessment included visual acuity (VA), central retinal thickness (CRT), slit-lamp biomicroscopy, and Goldmann tonometry for intraocular pressure (IOP) at 1, 6, and 12 months.

***Results*:**

Baseline mean VA was 47 (SD 18) letters improved to 55 (SD 17) letters (*P*=0.004) at 12 months. VA was improved in 16 eyes (57%), stabilised in 9 eyes (32%), and decreased in 3 eyes (11%). Seven eyes (25%) gained ≥15 letters, and 10 eyes (36%) gained >10 letters from baseline. The percentage of eyes achieved driving vision (≥70 Early Treatment Diabetic Retinopathy Study letters) was doubled from baseline 18 to 36% at 6 months and 32% at 12 months. Mean CRT decreased by 198 *μ*m from baseline 494 *μ*m (SD 191) to 296 *μ*m (SD 121) at 12 months (*P*<0.001). Two eyes received additional anti-vascular endothelial growth factor injections after 10 months. Complications: Raised IOP in three eyes (11%) controlled with IOP-lowering drops, vitreous haemorrhage in one eye and one endophthalmitis (1 year vision improved to 6/24).

***Conclusion*:**

Our real-world results show that the visual and the anatomical improvements achieved by a single ILUVIEN implant injection were maintained up to 12 months with minimal adjunctive therapy. IOP monitoring remains essential in ILUVIEN patients, although our study shows a relatively low risk of IOP elevation post ILUVIEN injection, even in existing controlled ocular hypertension. Our results demonstrate that ILUVIEN is an effective long-term option in treating chronic refractory DMO.

## Introduction

Diabetic macular oedema (DMO), as the common complication in diabetic retinopathy, is the leading cause of blindness in the working population among patients aged 20 to 70 years in developed countries.^1, 2^ The pathophysiology of DMO is a complex process with numerous biochemical and histopathological abnormalities whereby hyperglycaemia initiates molecular pathways leading to dilated capillaries, retinal microaneurysms, and loss of pericytes.^3^ This results in impairment of the blood–retinal barrier and increased vascular permeability, causing fluid to accumulate in retinal tissue.^4, 5, 6^ At early disease stages, vascular endothelial growth factor (VEGF) is the major driver of retinal vascular permeability change. However, a large number of physiological and molecular factors, including angiogenesis, inflammation, and oxidative stress, are involved in the pathogenesis of DMO.^5, 6, 7, 8^ The underlying pathogenesis is usually multifactorial, especially in chronic and refractory DMO.

Current treatment options of DMO include focal/grid macular laser photocoagulation and the use of intravitreal drugs depending on the localisation of DMO. Macular laser photocoagulation was the standard of care for over 30 years, but visual acuity (VA) gain was only modest.^9, 10, 11^ In recent years, intravitreal drugs targeted towards VEGF has become first-line therapy in foveal-involving DMO. However, the blockade of one single pathway may not represent an optimum treatment strategy as anti-VEGF do not suppress all the inflammatory cytokines and pathways involved in DMO and this may explain the need of frequent retreatment or insufficient response.

There has been interest in intravitreal corticosteroids, which are not only able to attenuate some of the effects driven by overexpression of VEGF but also reduce inflammation, which is recommended for DMO with insufficient response to anti-VEGF agents.^12^ The two licensed and approved corticosteroid implants in the United Kingdom for treating pseudophakic DMO are Ozurdex (a biodegradable 700 *μ*g of dexamethasone) and ILUVIEN (a non-biodegradable 0.2 *μ*g per day fluocinolone acetonide). Whilst the treatment effect of Ozurdex lasts for up to 6 months, the effect of ILUVIEN is up to three years.^13, 14, 15, 16^ While the efficacy and safety of ILUVIEN are well studied and established in clinical trials, there is however no long-term real-world published literature available on chronic DMO treated with ILUVIEN.^15, 16^ We report for the first time a larger series and a longer term of 12-month results on the efficacy and safety of chronic DMO treated with ILUVIEN in a real-world clinical practice in a tertiary referral eye centre in the United Kingdom.

## Materials and methods

Retrospective analysis of consecutive 28 eyes of 23 patients treated with ILUVIEN (0.2 *μ*g per day FAc) implant during the period from 2014 April to 2015 April. Data collection was from patients' medical records and included baseline characteristics, general and ocular history, and previous DMO treatment. Patients were examined before ILUVIEN treatment (baseline) and then regularly at 1 (for the detection of early intraocular pressure (IOP) change), 3, 6, 9, and 12 months post ILUVIEN implant. Standard ophthalmic assessment included VA (measured in Snellen), slit-lamp biomicroscopy, central retinal thickness (CRT) measurement using Topcon ocular coherence tomography SD-OCT (3D OCT-2000; Topcon Corporation, Tokyo, Japan) and Goldmann tonometry (Haag-Streit, Koeniz, Switzerland) for IOP.

Using a pre-agreed Microsoft Excel form data collection was from patients' medical records of visits at baseline, 1, 6, and 12 months post ILUVIEN implant and ±2 weeks was accepted as visit window. The mean number of follow-up visits in the first year was SD 4±1. The primary end point was the change in VA at 12 months. The other secondary outcomes assessed were the change in CRT, the change in IOP, adverse events, and the need for rescue treatments. Statistical analyses were performed using Wilcoxon's signed-rank test and the paired-sample *t*-test, with a level of *P*<0.05 being accepted as statistically significant using SPSS software (version 22.0; SPSS Inc., Chicago, IL, USA) programme. Snellen VA were converted into Early Treatment Diabetic Retinopathy Study (ETDRS) letter scores as described by Gregori *et al*,^17^ to facilitate statistical calculation.

The intravitreal procedure of ILUVIEN implant was performed in an aseptic theatre setting and postoperative chloramphenicol eye drops four times a day was advised for 1 week. Four eyes received ILUVIEN implant as a concurrent procedure with phacoemulsification and intraocular lens implant (eyes no. 5, 6, 8 and 28). As this analysis was part of our hospital clinical audit requirement, no ethical approval was needed. All patients received informed consent for investigations and procedures.

## Results

Demographic and baseline characteristics are listed in Table 1. Twenty-three patients were identified: 9 (39%) males and 14 (61%) females, with a mean age of 67 (SD 11) years; 19 (83%) and 4 (17%) had type II and type I diabetes mellitus, respectively.

Mean duration of DMO was 6 (SD 2) years before the ILUVIEN implant. Table 1 summarises various treatment modalities and frequencies each patient received before ILUVIEN treatment. Seven eyes (25%) had controlled ocular hypertension (OHT), two eyes (7%) had previous vitrectomy procedure, two eyes (21%) had existing epiretinal membrane and two eyes (7%) also had viteromacular traction.

### VA results

**Figure 1:**
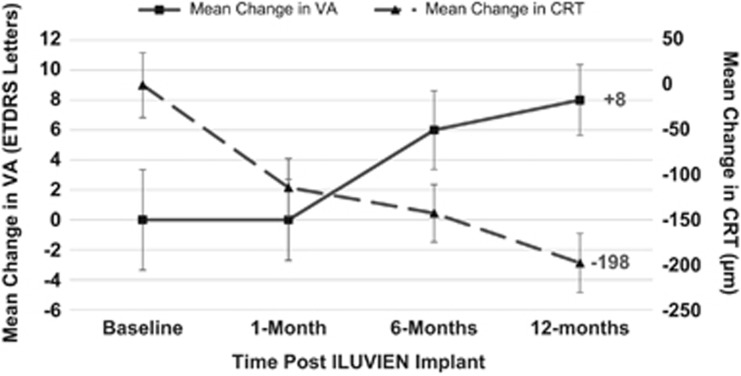
Mean changes in VA and CRT compared with baseline.

Baseline mean VA was 47 (SD 18) letters, ranged from −4 to 76 letters (Snellen equivalent 1/60 to 6/9), improved to 55 (SD 17) letters at 12 months (Figure 1). Compared with baseline VA, mean VA change was statistically significant at months 6 and 12 but not at first month (Table 2). However, eyes with poor baseline VA because of chronic DMO (ETDRS letter score of ≤35, Snellen equivalent 6/60 or worse) achieved the greatest mean VA improvement at 12 months (+16 letters), and the ‘relatively good' baseline VA group achieved the least VA gain (Table 3).

**Figure 2:**
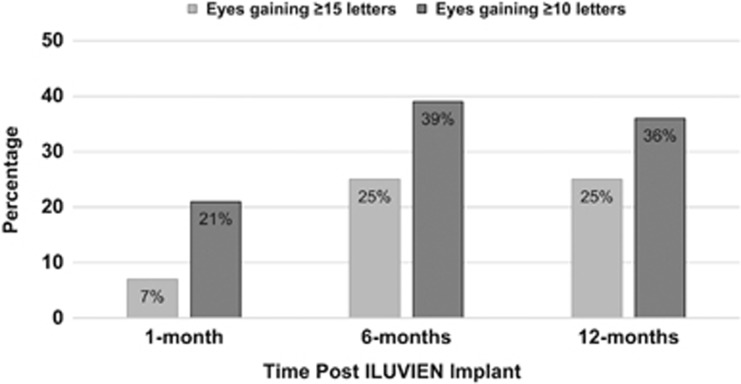
Visual outcome post ILUVIEN implant.

At 12 months VA improved in 16 eyes (57%), stabilised in 9 eyes (32%), and decreased in 3 eyes (11%), the percentage of eyes which gained ≥15 letters was 25% (7 eyes), and 10 eyes (36%) gained >10 letters from baseline (Figure 2). VA decreased only in 3 eyes (eyes no. 3, 5, and 16) at 12 months, with none of them losing more than 11 letters. VA of 35 letters or less was reported in 7 eyes (25%) at 12 months compared with 12 eyes (43%) at baseline.

### CRT results

Mean CRT reduction was statistically significant in all visits compared with the baseline CRT of 494 *μ*m (SD 191); the greatest reduction was by 198 *μ*m (*P*<0.001) at 12 months (Figure 1 and Table 2). At baseline, only 6 eyes (21%) had CRT <300 *μ*m and 19 eyes (68%) had CRT >400 *μ*m, which reported the greatest reduction in CRT at 12 months. At month 12, CRT reduction was evident in 24 eyes (86%) and increased in 4 eyes (14%).

### Adverse events and rescue treatments results

Following ILUVIEN injection, only 3 eyes (11%) had raised IOP ≥10 mmHg at 6 months in eye no. 1, day 3 in eye no.16, and at 3 months in eye no. 21. Vitreous haemorrhage was reported in one eye (4% eye no. 3) after injection, which resolved over few weeks. A poorly controlled diabetic had culture-positive endophthalmitis (eye no.16) received intensive antibiotics treatment and recovered well to 6/24 vision at 1 year. Only two eyes (7%) received rescue treatment in the first year with Ranibizumab injection (at 10 months in eye no. 22 and at 12 months in eye no. 4).

## Discussion

Our real-world 12-month results demonstrated compatible and potentially more favourable visual and anatomical outcomes than the pivotal FAME clinical trial at month 12: our study has a mean improvement of +8 letters in VA from baseline compared with +4.9 letters in the FAME trial (also based on chronic DMO subgroup).^18^ Twenty five per cent in our study gained ≥15 ETDRS letters *vs* 23.4% in the FAME trial.^15^ Our study has also shown a greater reduction or improvement of mean change in CRT of minus 198 *μ*m *vs* minus 156.6 *μ*m in the FAME trial.^15^ The favourable visual outcome in our study may possibly be explained by a better baseline VA in the FAME trial patients (hence less chance of ‘greater improvement'). In addition, most patients in our study had received previous anti-VEGF treatment unlike patients in the FAME trial.

In our cohort with relatively small numbers of study patients, we found no significant safety concern between eyes received ILUVIEN implant as concurrent procedure with phacoemulsification surgery (eyes no. 5, 6, 8, and 28), and eyes received ILUVIEN alone. Arguably, VA improvement in these four patients could be related to cataract surgery, but the reduction of CRT however is more suggestive of the efficacy of ILUVIEN, as more often DMO are likely to worsen or unchanged following cataract surgery: CRT at baseline *vs* CRT at 12 months were 825→350 (eye no. 5), 222→240 (eye no. 6), 426→218 (eye no. 8), and 475→246 (eye no. 28). Interestingly, the end statistical results remain unchanged if these four eyes were not included in the main analysis (Table 2). Our real-world study hence suggests the option of ILUVIEN implant may be delivered safely and at the earliest benefit, as a concurrent procedure with the cataract surgery.

As our practice serves a population with diverse ethnicity (Table 1), our study included 30.5% Caucasian, 26% Afro-Caribbean/Africans, and 43.5% Asian. There were also no significant outcome differences among the ethnic groups in this study; however, larger studies would be needed to address these interesting subanalyses.

The percentage of eyes achieved driving vision (70 ETDRS letters or more) was doubled from baseline 18 to 36% at 6 months and 32% at 12 months. This is indeed a very significant and welcome result, especially for the younger diabetics who lead active working lives and are keen to maintain driving. Our study has also shown much reduced clinic visits needed in the first year with a mean of 4±SD1 visits; this is beneficial and appreciated by both patients and helps to relieve the overstretched hospital service from frequent monthly anti-VEGF treatment.^19^

### Secondary OHT with ILUVIEN

Clinicians have indeed been reluctant to apply intraocular or periocular steroid as the first-line treatment for DMO based on the well-known adverse effect of OHT or secondary glaucoma up to 30–40%.^15, 19, 20, 21, 22, 23, 24, 25^ In our study, 11% (3 eyes) required initiation of IOP-lowering drops compared with 18.4% in the ILUVIEN Registry Safety Study (IRISS) and 22% in the FAME trial.^26^ The difference is possibly because of the small number of eyes in this study, and the other possibility is that patients already on IOP-lowering drops were excluded from the FAME trial; however, in our study, 25% (7 eyes) were known to have controlled OHT on topical treatment. None of our study eyes needed further invasive glaucoma procedures or surgery in the first year, although eye no. 21 had received selective laser trabeculoplasty at month 16 to maintain IOP control without IOP-lowering drops.

We are not aware of any published literature correlating risk and effect of ILUVIEN implant on patients with OHT. Our study shows the risk of IOP elevation secondary to ILUVIEN implant injection was not higher in patients with controlled OHT compared with patients without OHT. Nevertheless, a recent review of current literature proposed an algorithm to provide guidance for the monitoring and management of IOP following treatments with corticosteroids in DMO, suggesting imaging of optic nerve head fibres and visual field test for at-risk patients before corticosteroid intravitreal injections.^27^ Intraocular steroid is also associated with additional risk of cataract formation, but it was not shown in this study as ILUVIEN is approved to be used only in pseudophakic DMO eyes in the United Kingdom.

### Rescue treatment

In this study, two eyes received additional anti-VEGF injections: eye no. 22 received Ranibizumab at month 10 when CRT did not improve and subsequent further VA reduction; eye no. 4 had initial good response with ILUVIEN with significant VA improvement, but CRT increased after month 6 and hence received intravitreal Ranibizumab injection at month 12. There is no guidance in any existing published literature (based on short-term results) on ‘rescue therapy', including any guidance from the manufacturer. In our series, the decision on ‘rescue therapy' was offered when recorded VA deterioration (5 letters or more, equivalent to one Snellen line) was due to increase in CRT (and CRT was ≥400 *μ*m). Our paper is the first to suggest the window and safety of ‘rescue therapy' after ILUVIEN implant, which is close to 1-year window. Ranibizumab was the choice of ‘rescue therapy' as at the time of study, it was the only licenced anti-VEGF available.

In addition, this study had included two vitrectomised eyes (eyes no. 15 and 17), although the number is too small to draw any definitive conclusion, both vitrectomised eyes have shown a longer lasting favourable CRT reduction without any additional rescue therapy. One of the eyes (eye no.15) in this series had been reported earlier in published case-report.^28^

Larger real-life outcomes data may explain more the role of macular laser and anti-VEGF as rescue treatment and when to designate a patient as inadequate responder. The main strengths of our study are the availability of longer term result up to 12 months and a larger series representation of ‘real-world' experience in managing chronic struggling DMO and it shows the effect of ILUVIEN implant on patients with OHT. Our study limitations are its retrospective nature with no comparators and using non-refracted Snellen VA scores.

## Conclusion

Our real-world single-centre results demonstrate that ILUVIEN offers a significant long-term benefit for patients with chronic DMO inadequately responsive to other available therapies. Favourable visual and anatomical improvements were achieved by a single ILUVIEN implant injection in the first year with minimal adjunctive therapy. Our study also shows relatively low risk of raised IOP following ILUVIEN injection in patients with controlled OHT. However, IOP monitoring remains essential in patients receiving ILUVIEN implant. Longer and larger real-world data may provide a comprehensive long-term management strategy.


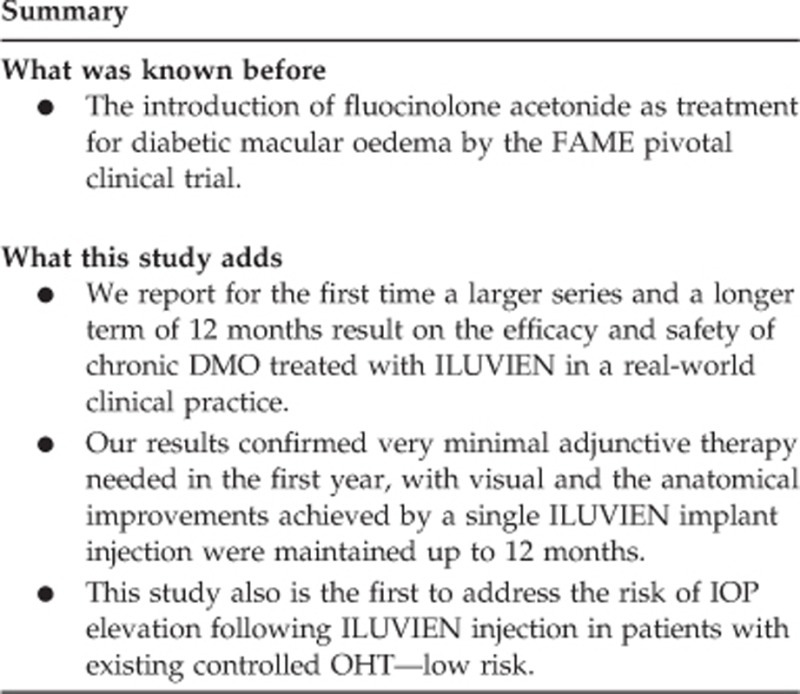


## Figures and Tables

**Table 1:** Demographic and baseline characteristics

*Patient number*	*Gender*	*Age (years)*	*Ethnicity*	*Diabetes type*	*Eye number*	*Laterality left*/*right*	*DR grade*	*DMO duration (years)*	*Previous treatment for DMO*					*IOP-lowering eye drops*
									*Macular laser /vitrectomised*	*IVB*	*IVR*	*IVTA*	*Ozurdex*	
1	M	55	Bangladeshi	I	1	L	R2M1P1	7	Focal 1x	0	6x	0	0	N
2	F	82	Caribbean	II	2	R	R3M1P1	7	Focal 3x, grid 2x	2x	0	1x	0	N
					3	L	R2M1P1	7	Focal 1x, grid 2x	0	0	2x	0	N
3	F	81	Indian	II	4	R	R1M1P1	6	Focal 3x, grid 1x	5x	6x	0	0	N
4	F	68	Indian	II	5	R	R2M1P1	7	Focal 4x, grid 2x	3x	6x	0	1x	N
					6	L	R2M1P1	7	Focal 4x, grid 3x	3x	0	0	1x	N
5	M	71	Bangladeshi	II	7	L	R1M1P0	2	No	0	3x	0	0	N
6	M	70	Caucasian	II	8	R	R3M1P1	6	Focal 1x, grid 2x	6x	6x	1x	0	N
7	M	57	Caucasian	II	9	R	R3M1P1	6	Focal 1x	3x	0	6x	0	Cosopt
					10	L	R3M1P1	6	No	3x	0	6x	0	Cosopt
8	F	74	Pakistani	II	11	R	R1M1P1	6	Focal 4x	0	5x	0	0	N
9	F	79	Caribbean	II	12	R	R3M1P1	14	Focal 4x, grid 3x	6x	2x	0	0	N
10	M	63	Caribbean	II	13	R	R1M1P1	11	Focal 5x	3x	5x	0	0	Xalatan
11	M	48	Caucasian	I	14	L	R3M1P1	5	Focal 1x	11x	0	7x	0	N
					15	R	R3M1P1	6	Grid 1x, vitrectomised	12x	0	4x	1x	N
12	F	66	Chinese	II	16	R	R3M1P1	4	Grid 1x	8x	2x	1x	0	N
13	F	59	Indian	II	17	L	R1M1P1	5	Focal 2x, vitrectomised	4x	3x	2x	0	N
14	F	70	Pakistani	II	18	R	R2M1P1	5	Focal 3x	0	0	0	0	N
15	F	66	Caucasian	II	19	R	R1M1P1	4	Focal 1x, grid 2x	3x	0	2x	0	N
16	M	59	Caucasian	II	20	R	R1M1P0	3	No	7x	6x	0	0	N
17	F	53	Caucasian	I	21	L	R3M1P1	5	Grid 1x	7x	3x	2x	0	Tiopex
18	M	55	Indian	I	22	L	R3M1P1	4	Focal 1x, grid 1x	2x	3x	1x	0	Azopt
19	F	59	Other Black background	II	23	R	R3M1P1	5	Focal 3x, grid 2x	3x	9x	8x	0	N
20	F	82	Caribbean	II	24	R	R1M1P1	10	Focal 1x, grid 7x	6x	0	0	0	Cosopt
21	F	82	Caribbean	II	25	R	R1M1P1	5	Focal 1x	5x	7x	2x	0	N
					26	L	R1M1P1	3	Focal 1x	3x	7x	2x	0	N
22	F	68	Bangladeshi	II	27	R	R3M1P1	6	Focal 1x	3x	3x	0	0	N
23	M	68	Caucasian	II	28	R	R1M1P1	5	Focal 1x	10x	9x	5x	0	Latanoprost

Abbreviations: DMO, diabetic macular oedema; DR, diabetic retinopathy; F, female; IOP, intraocular pressure; IVB, intravitreal bevacizumab; IVR, intravitreal ranibizumab; IVTA, intravitreal triamcinolone acetonide; L, left; M, male; N, none; R, right; RMP, retinopathy, maculopathy, photocoagulation.

**Table 2:** Changes in baseline vision and central retinal thickness

*Number of eyes*^a^	*Mean VA in letters*	*Mean CRT (*μ*m)*	*Eyes with VA ≥70 letters (%)*
*Baseline*			
28	47 (SD 18)	494 (SD 191)	5/28 (18%)
24	46 (SD 19)	495 (SD 191)	

*One month post ILUVIEN*			
28	47 (SD 21) *P*=0.913	323 (SD 143) *P*=0.003	6/28 (21%)
24	46 (SD 21) *P*=0.797	379 (SD 157) *P*=0.018	

*6 months post ILUVIEN*			
28	53 (SD 17) *P*=0.021	351 (SD 149) *P*<0.001	10/28 (36%)
24	53 (SD 17) *P*=0.009	358 (SD 161) *P*=0.002	
			
*12 months post ILUVIEN*			
28	55 (SD 17) *P*=0.004	296 (SD 121) *P*<0.001	9/28 (32%)
24	54 (SD 17) *P*=0.004	301 (SD 131) *P*<0.001	

Abbreviations: CRT, central retinal thickness; SD, standard deviation; VA, visual acuity in Early Treatment Diabetic Retinopathy Study (ETDRS) letter score.

a Subanalysis of 24 eyes—excluded eyes underwent concurrent procedures of ILUVIEN with phacoemulsification.

**Table 3:** Mean change in vision at 12 months for different baseline visual acuity

*Baseline VA*	*Number of eyes*	*Mean change in VA compared with baseline*
≥70 letters (Snellen 6/12 or better)	5	+2 letters
36–69 letters (Snellen >6/12 to <6/60)	11	+12 letters
≤35 letters (Snellen 6/60 or worse)	12	+16 letters

Abbreviation: VA, visual acuity in Early Treatment Diabetic Retinopathy Study (ETDRS) letter score.
